# Determinants of victimization in patients with severe mental illness: results from a nation-wide cross-sectional survey in the Netherlands

**DOI:** 10.3389/fpsyt.2025.1511841

**Published:** 2025-03-17

**Authors:** Astrid M. Kamperman, Milan Zarchev, Jens Henrichs, Sten P. Willemsen, Emmanuel M. E. H. Lesaffre, Wilma E. Swildens, Yolanda Nijssen, Hans Kroon, Anneke D. J. F. van Schaik, Mark van der Gaag, Philippe A. E. G. Delespaul, Jaap van Weeghel, Dike van de Mheen, Stefan Bogaerts, Cornelis Lambert Mulder

**Affiliations:** ^1^ Department of Psychiatry, Epidemiological and Social Psychiatric Research Institute, Erasmus Medical Center (MC) University Medical Center, Rotterdam, Netherlands; ^2^ Midwifery Science, Amsterdam University Medical Center (UMC), location Vrije Universiteit Amsterdam, Amsterdam, Netherlands; ^3^ Midwifery Academy Amsterdam Groningen, Inholland University of Applied Sciences, Amsterdam, Netherlands; ^4^ Mental Health, Amsterdam Public Health, Amsterdam, Netherlands; ^5^ Department of General Practice and Elderly Care Medicine, University of Groningen, University Medical Center Groningen, Groningen, Netherlands; ^6^ Department of Biostatistics, Erasmus Medical Center (MC) University Medical Center, Rotterdam, Netherlands; ^7^ Department of Epidemiology, Erasmus Medical Center (MC) University Medical Center, Rotterdam, Netherlands; ^8^ Interuniversity Institute for Biostatistics and Statistical Bioinfomatics (I-Biostat), KU-Leuven, Leuven, Belgium; ^9^ Altrecht Institute for Mental Health Care, Utrecht, Netherlands; ^10^ Inholland University of Applied Sciences, Amsterdam, Netherlands; ^11^ Parnassia Psychiatric Institute, Den Haag, Netherlands; ^12^ Amsterdam University of Applied Sciences, Amsterdam, Netherlands; ^13^ Tranzo Scientific Center for Care and Welbeing, Department of Social and Behavioral Sciences, Tilburg University, Tilburg, Netherlands; ^14^ Department of Reintegration and Community Care, Trimbos Institute, Netherlands Institute of Mental Health and Addiction, Utrecht, Netherlands; ^15^ Department of Psychiatry, Amsterdam University Medical Center (UMC), Amsterdam Public Health Research Institute, Vrije Universiteit (VU) University Medical Center, Amsterdam, Netherlands; ^16^ GGZ inGeest Mental Health Care, Amsterdam, Netherlands; ^17^ Department of Clinical Psychology, Vrije Universiteit (VU) University and Amsterdam Public Health Research Institute, Amsterdam, Netherlands; ^18^ Department of Psychiatry and Neuropsychology, Faculty of Health, Medicine and Life Sciences, School for Mental Health and Neuroscience (MHeNs), Maastricht University, Maastricht, Netherlands; ^19^ Mondriaan Mental Health Centre, Maastricht, Netherlands; ^20^ Department of Developmental Psychology, Tilburg University, Tilburg, Netherlands; ^21^ Fivoor Science and Treatment Innovation, Rotterdam, Netherlands

**Keywords:** victimization, violence, crime, determinants, psychiatric patients, epidemiology

## Abstract

**Objective:**

To examine determinants of the prevalence and frequency of criminal victimization (i.e. both personal and property crime victimization) in outpatients with severe mental illness.

**Methods:**

Data was collected using a multisite epidemiological survey including a random sample of 956 adult outpatients with SMI. Data were collected between 2010 and 2012. Data on 12-month victimization prevalence and frequency were obtained using the victimization scale of the Dutch Crime and Victimization Survey. Demographic characteristics, clinical determinants, e.g., clinical diagnosis, psychosocial functioning, drug use and alcohol abuse over the past 12 months, co-morbid PTSD diagnosis, and victimological determinants e.g. physical abuse, physical neglect and sexual abuse in childhood, perpetration of violence over the past 12-months, and anger disposition were assessed as determinants. Univariable and multivariable hurdle regression analyses were conducted to test associations of the potential determinants with victimization prevalence and frequency.

**Results:**

Twelve-month prevalence rates of personal and property crime were 19% and 28%, respectively. Clinical characteristics were more pronounced regarding personal crime victimization. In the multivariable model, presence of psychotic disorder, drug use, childhood physical and sexual abuse, and recent violent perpetration were associated with the 12-month prevalence or frequency rate of personal crime victimization. Native Dutch and divorced patients were more at risk as well. Next to this being employed, poor social functioning, having perpetrated a violent crime, as well as alcohol abuse and recent drug use were all significantly related to property crime prevalence or frequency rate in the multivariable model. In absolute terms, the effect sizes observed tended to be moderate to small. The multivariate models, however, explained the outcome variance moderately well (Nagelkerke’s pseudo R^2^ = 25.0 - 27.9%.

**Conclusions:**

Clinicians should be aware of the high risk of victimization among their patients with severe mental illness. Particular attention should be devoted to people with substance use histories and perpetrators of violence, since they are also at an increased risk of being victims as well.

## 1 Introduction

Outpatients with severe mental illness (SMI), including bipolar, depressive or psychotic disorders, have a higher risk of falling victim to a crime than the general population (1–3).

As an example, a randomized survey in Chicago (*n* = 936) demonstrated that adult outpatients with SMI had an 11-times higher prevalence of personal crime victimization compared to the general population (3). Crime victimization refers to the involuntary exposure of criminal acts which can be distinguished in two categories: (a) personal crime, which includes victimization events assault, battery, and rape; and (b) property crime which results in theft, larceny or burglary of belongings (3, 4).

Crime victimization is associated with psychopathology (e.g., posttraumatic stress disorder (PTSD), psychotic disorders, anxiety and substance abuse), stigmatization and perpetration of violence (1, 5–9). Moreover, victimization among psychiatric patients is related to exacerbation of existing mental problems, increased service use of health services and suboptimal treatment results (9–11). These figures are alarming and raise the urgent question of identifying risk factors of crime victimization. Risk factors can be broadly categorized as sociodemographic (e.g. the victim’s sex, housing, or socio-economic status), clinical (e.g. mental health problems, drug and alcohol abuse, or social functioning) and victimological (e.g. childhood neglect or abuse, crime perpetration) (3). Such information is crucial, because it can be used for the development or fine-tuning of prevention and intervention programs to address (re-)victimization of outpatients with SMI and its consequences.

Deinstitutionalization of psychiatric care may be a key factor in the increase of reported crime victimization among patients with SMI. Although, compared to the US, deinstitutionalization has been less drastic in European countries, such as the Netherlands (12–14), the majority of the Dutch SMI patients now receive less care from 24-hour hospital services and are instead living in the community. Among persons with SMI, deinstitutionalization may have potentially increased the rates of homelessness, which constitutes an important risk factor of victimization (15–20). As persons with SMI are more often confronted with other adverse conditions (e.g., unemployment, poverty, being a member of an ethnic minority, and conflict prone relationships) these factors may also constitute additional high-risks for victimization (21–23). It is unknown, however, whether victimization has genuinely increased in numbers or instead spilled outside institutional care where crime incidents could be neglected and thus have remained unreported (24). In addition, because of psychiatric problems, including substance abuse, poor reality testing and judgment, reduced social skills as well as impaired emotional regulation, persons with SMI are likely targets to be at an increased risk of victimization (25–29). Finally, persons with SMI have often experienced violent, sexual and/or emotional victimization in childhood, which may make them more vulnerable for victimization in adulthood due to learned helplessness (30–33).

The above lines of research suggest that various socio-demographic adversities, psychiatric problems, and childhood experiences of victimization may contribute to the increased risk of crime victimization among adult outpatients with SMI. However, systematic research simultaneously studying multiple risk factors of crime victimization among outpatients with SMI based on large-scale representative samples is lacking. Some previous studies have looked at specific clinical predictors of victimization for people with SMI, such as the duration and severity of illness, hospitalizations and history of trauma (3, 35). No studies so far have offered a broad perspective on how broader risk factors coalesce to increase the risk of victimization. This leaves an important research gap for holistically identifying systems of risk factors which predict victimization using a multivariable approach.

The current study is a nation-wide randomized multi-site epidemiological survey on victimization among SMI outpatients in the Netherlands. It is the first nationwide multi-site epidemiological study in Europe on assessing both the 12-month prevalence and 12-month frequency rates of crime victimization among adult outpatients with SMI. It includes additional information on various potential criminal victimization, In the current study we aim to estimate the impact of potential demographic, clinical, and victimological determinants on crime victimization prevalence and frequency. We differentiate between personal and property crimes.

## 2 Methods

### 2.1 Design

The current study is embedded in the Victimization in Psychiatric Patients (ViPP) study, a cross-sectional epidemiological survey of a large random community sample of 956 patients with SMI in the Netherlands (36). Participants were randomly selected from the caseload of six Mental Health Care (MHC) organizations in the Netherlands providing outpatient care to patients suffering from SMI. The organizations are located in both urban and rural areas of the Netherlands and provide care to a range of 240 to 2000 patients with chronic psychotic, bipolar or major depressive disorders. The patient populations at the participating MHC organizations are representative of the SMI patient population in the Netherlands (37–39). Participants were enrolled between December 2010 and April 2012. Written informed consent was obtained from all participants. The study was approved by the Medical Ethics Committee of the Erasmus MC, Rotterdam (MEC-2010-232) in accordance with the Declaration of Helsinki and Dutch Act of Medical Research involving Humans (WMO Act).

### 2.2 Participants

Eligible for the study were all people aged between 18 and 65, being outpatients of one out of six of the participating MHC organizations. A random sample of 3336 eligible outpatients was selected from the patient administration system of each participating site. In- and exclusion criteria were checked by the treating clinician. Excluded were patients with insufficient command of the Dutch language. Those incarcerated in prison or admitted to an acute hospital service and unable to answer study questions due to their psychiatric condition (severe symptomatology, psycho-organic disorders, high levels of aggression or cognitive impairments) were also excluded. This resulted in a sample of 2572 eligible patients who received an invitation letter, and were subsequently contacted by the research team. After signing informed consent the patient was included in the study. Full details on the recruitment has been published previously (36). Flowchart of the recruitment process can be found in the **Appendix Figure A (S1)**.

### 2.3 Procedures

Data on crime victimization and determinants were obtained in a structured, computer-assisted face-to-face interview. Respondents were paid 20 Euro in cash at the end of the interview. The patient’s interview took 75 minutes on average (range: 40-160 minutes) and was carried out at the respondent’s discretion in his or her home or at the MHC organization.

### 2.4 Interviewers

Data on crime victimization and determinants of crime victimization were collected by interviewers who were master’s level social scientists, e.g., psychologists and sociologists. These interviewers were trained in conducting the structured computer-assisted interview, and in interviewing skills adapted to persons with SMI by senior researchers experienced with the study research population and an actor. An experienced interview coordinator with a master’s level in social science in collaboration with the researchers (AMK, JH) supervised the interviewers and monitored the quality of the interviews.

### 2.5 Instruments

#### 2.5.1 Victimization

Twelve-month prevalence of crime victimization and the number of incidents were assessed using the crime victimization scale of the Dutch Crime and Victimization Survey [in Dutch: ‘*Integrale Veiligheidsmonitor’*(IVM)] (40). The IVM crime victimization scale strongly resembles the International Crime Victimization Survey (41). The IVM consists of 14 screening questions on various types of property crime, personal crime and vandalism. For each reported incident in the preceding 12 months detailed information on the time and number of incidents, setting and perpetrator was assessed. To minimalize the effect of telescoping, the respondents were asked to recall incidents over the past five years, before recalling incidents over the past 12 months (4, 40).

The IVM assessed the following crime victimization categories: Personal crime victimization consisting of sexual harassment or assault, being threatened with violence, and threatened with physical assault; Property crime consisting of burglary, attempted burglary, bike theft, pick-pocketing, robbery, and theft (other). The total crime category consists of burglary, attempted burglary, bicycle theft, pick-pocketing, robbery, theft, vandalism (other), sexual harassment or assault, threatened with violence, physical assault, and crime (other). Since car ownership has low prevalence in the current sample (42), car-related crimes (car theft, theft from car and vandalism of car; n = 5 crime thefts in the current sample) are included in the crime categories used for sensitivity analysis only.

### 2.6 Determinants

#### 2.6.1 Social demographic characteristics

Socio- demographic characteristics included gender, age, ethnicity, marital and employment status, educational level, housing status, and urbanicity. Following the definition of the Dutch government (43) ethnicity was classified on the basis of country of birth and his/her parents’ country of birth. If the parents were born in different countries, the mother’s country of birth prevails. Information about the population density on a postal code level for 2010 was obtained from the national bureau of statistics and matched to each participants. All other information was obtained via the interview.

#### 2.6.2 Clinical diagnosis

The primary psychiatric diagnosis, i.e., psychotic, bipolar or major depressive disorder was extracted from the electronic patient files (EPF). Commonly this is a diagnosis set by the psychiatrist or clinical psychologist at the start of the treatment/intake, and updated by the primary clinician. Diagnosis extracted from the EPF were cross-checked by the primary clinician at the start of the study.

#### 2.6.3 Psychosocial functioning

Psychosocial functioning was assessed using the Health of the Nation Outcome Scales (HoNOS) (44), an observational instrument. The HoNOS consists of 12 items, covering a range of health and social domains, i.e. overactive, aggressive or agitated behavior; non-accidental self-injury; problem drinking or drug taking; cognitive problems; physical illness or disability; problems associated with hallucinations and delusions; problems with depressed mood; other mental and behavioral problems; problems with relationships; problems with activities of daily living; problems with living conditions; problems with occupation and activities. The items are scored on a 5-point scale, ranging from ‘no problem’ to ‘(very) severe problem’. In accordance with HoNOS instruction, the rating was based on all information available to the rater and was related to the most severe problem that occurred during the period rated (usually the two weeks leading up to the point of rating). HoNOS questionnaires were scored by the primary clinician, and shared with the research team. The reliability of the HoNOS in a Dutch population was sufficient (Cronbach α =0.78), and so were divergent and concurrent validity (45). The cut-off score of 9 or higher was used to dichotomize the total score in two categories: mild problems and moderate to severe problems in psychosocial functioning (45, 46), which coincide with the median score in this sample.

#### 2.6.4 Substance abuse

Substance abuse was assessed using the Dutch version of the 12-month drug and alcohol use questionnaire of the European Monitoring Centre for drugs and Drugs Addiction (EMCDDA) (47). Regarding alcohol use, frequency and quantity were assessed. For this study, we operationalized alcohol abuse as at least one episode of heavy drinking or binge drinking (e.g. more than 6 consumptions at one occasion) during the past 6 months in line with the definitions used by the Centres for Disease Control and Prevention and World Health Organization (48, 49). With regard to drug use, type and recency were assessed. Drugs use was operationalized as using one or more types of drugs, or using medication without a doctor’s prescription.

#### 2.6.5 Co-morbid PTSD

Symptoms of posttraumatic stress disorder (PTSD) were assessed using the Self-Rating Inventory for Posttraumatic stress Disorder (SRIPD) (50). The questionnaire consisted of 22 items, reflecting the 17 PTSD symptoms according to DSM-IV. The items were scored on a 4-point Likert scale, ranging from ‘no problem’ to ‘very severe problem’. A score above 52 points was interpreted as the presence of PTSD (50, 51). Sensitivity was estimated as 86% and specificity was 71%. Reliability is good (Cronbach α ranges from 0.90 to 0.94); construct validity is satisfactory (50).

#### 2.6.6 Childhood victimization

Childhood victimization was assessed using three scales of the short form of the Childhood Trauma Questionnaire (CTQ) (52). The CTQ is a tool to detect histories of maltreatment. Items on the CTQ assessed experiences in childhood and adolescence which were rated on a 5-point Likert-type scale with response options ranging from ‘never true’ to ‘very often true’. The subscales physical abuse (5 items), physical neglect (5 items), and sexual abuse (5 items) were included in this study. Psychometric research showed that the construct and criterion-related validity of the CTQ was satisfactory; test-retest reliability of the CTQ subscales ranged from.79 to.86, and internal consistency coefficients ranged from.66 to.92 (52). The scores on the subscales were dichotomized in ‘no experiences’, and ‘one or more experiences’.

#### 2.6.7 Perpetration of violence

Perpetration of physical violence over the past 12-months was assessed by the physical assault subscale (12 items) of the Conflict Tactics Scale short form (CTV2) (53). The items assessed mild and severe experiences of violent perpetration and were answered on a 6-point scale with response options ranging from ‘once a year’ to ‘more than 20 times a year’. Response options for ‘never happened’, and ‘did not happen in the past year, but it did happen before’, were also included. The score was dichotomized in ‘no experiences’, and ‘one or more experiences’.

#### 2.6.8 Dispositional anger

The Dimensions of Anger Reactions scale (DAR) (54) was used to assess trait anger. The DAR consisted of seven items. The scale assessed anger, frequency, intensity, duration, antagonistic expression, and impairment of work performance, interpersonal relationships, and personal health. In the Dutch version, the items were scored on a 5-point scale ranging from 0 ‘not at all’ to 4’very much’ (55). Higher scores reflected higher dispositional anger. Test-retest reliability in a Dutch population was good (r=0.84); construct validity was satisfactory (55). Internal consistency was good (Cronbach’s alpha = 0.79). The score was dichotomized into a low and a high dispositional anger category using a score of 12 as the cut-off point (56).

### 2.7 Statistical analysis

Hurdle regression analyses were conducted to separately estimate the effects of factors associated with victimization prevalence and victimization frequency over the previous 12 months (57). This model is estimated using logistic regression to model the dichotomous outcome (any vs no victimization incident, i.e., the zero component), and a zero-truncated negative binomial regression for the count outcome (the number of victimization incidents i.e., the count component). Separate models were estimated for personal crime and for property crime victimization outcomes. We used R software (version 4.02) (58) and made use of the ‘pscl’ package (59, 60) for estimating the parameters of the hurdle models.

We first assessed the univariable impact of all determinants on victimization. Second, we entered all determinants into a multivariable model (full model), regardless of the univariable significance obtained in the first step. Next, we excluded variables based on two rules: a significant Likelihood Ratio-test of the reduced model fit at the 5% level and a lack of coefficient change (Δ β >20%) for any of the main determinants (gender, urbanicity and level of psychosocial functioning). We started from least to most significant variable in the full-model (61). The main determinants remained in the model, irrespective of significance or coefficient change. In a last step, the final reduced model was re-estimated using the Sandwich estimation method as implemented in the ‘Sandwich’ package^1^ (62). Continuous determinants were dichotomized to improve interpretation of the coefficients. We report on Nagelkerke’s pseudo-R^2^ as a measure of effect size and use contemporary rules of thumbs to interpret its magnitude (63).

Seven variables accounted for missing data: urbanicity (n=2; 0.5% of cases), alcohol abuse (n=10; 1.0% of cases), dispositional anger (n=10; 1.0% of cases), co-morbid PTSD (n=11; 1.2% of cases), perpetration of violence (n=13; 1.4% of cases), housing (n=29; 3.0% of cases), psychosocial functioning (n=260; 27% of cases).Missing values were imputed under the assumption of missing at random using a multiple imputation (five imputed datasets) as implemented in the ‘mice’ package (64). We report on pooled estimates, calculated using the ‘mitools’ package (65, 66).

Multicollinearity between determinant variables was assessed using variance inflation factors (VIF>2.0) as implemented in the ‘car’ package (67) and none were found to be above the threshold. Seven cases (0.7% of the total sample) were identified as outliers, reporting extremely high numbers of incidents (>65 incidents), and were removed additionally from the analyses on determinants of victimization frequency. With regards to the other socio-demographic and clinical characteristics, we observed strong similarities between the outlying and the non-outlying cases (see **Table 1** and **Appendix Table B (S2)**).

**Table 1:** Socio-demographic and clinical characteristics of the SMI patients in the sample.

		Full Sample (N=956)	Outliers (N=7)	Sample when outliers removed (N=949)	Imputed Sample (N=949)	Imputed cases N (%)
Sex	Male	608 (63.6%)	5 (71.4%)	603 (63.5%)	603 (63.5%)	
	Female	348 (36.4%	2 (28.6%)	346 (36.5%)	346 (36.5%)	
Age	18–30 yr.	102 (10.7%)	–	102 (10.7%)	102 (10.7%)	
	31-40 yr.	232 (24.3%)	–	231 (24.4%)	231 (24.4%)	
	41-50 yr.	305 (31.9%)	4 (57.1%)	301 (31.7%)	301 (31.7%)	
	51-65 yr.	317 (33.2%)	3 (42.9%)	314 (3.1%)	314 (3.1%)	
Ethnicity	Dutch native	587 (61.4%)	4 (57.1%)	583 (61.4%)	583 (61.4%)	
	Non-native	369 (38.6%)	3 (42.9%)	366 (38.6%)	366 (38.6%)	
Marital status	Single	550 (57.5%)	5 (71.4%)	545 (57.4%)	545 (57.4%)	
	Married/committed relationship	240 (25.1%)	2 (28.6%)	238 (25.1%)	238 (25.1%)	
	Divorced/widowed	166 (17.4%)	–	166 (17.5%)	166 (17.5%)	
Education	No/primary	217 (22.7%)	2 (28.6%)	215 (22.7%)	215 (22.7%)	
	Basic vocational	324 (33.9%)	3 (42.9%)	321 (33.8%)	321 (33.8%)	
	Intermediate vocational	268 (28.0%)	1 (14.3%)	267 (28.1%)	267 (28.1%)	
	High vocational/academic	147 (15.4%)	1 (14.3%)	146 (15.4%)	146 (15.4%)	
Employment	Yes	139 (14.5%)	1 (14.3%)	138 (14.5%)	138 (14.5%)	
	No	817 (85.5%)	6 (85.7%)	811 (85.5%)	811 (85.5%)	
Housing	Sheltered housing	196 (20.5%)	2 (28.6%)	194 (20.4%)	*200 (21.1%)*	*29 (3.0)*
	Single household	489 (51.2%)	3 (42.9%)	486 (51.2%)	*502 (52.9%)*	
	Family household	242 (25.3%)	1 (14.3%)	241 (25.4%	*247 (26.0%)*	
	Missing	29 (3.0%)	1 (14.3%)	28 (3.0%)	–	
Urbanicity	> 2500 inh./km^2^	882 (92.3%)	6 (85.7%)	876 (92.3%)	*878 (92.5%)*	*2 (0.5)*
	≤ 2500 inh./km^2^	72 (7.5%)	1 (14.3%)	71 (7.4%)	*71 (7.5%)*	
	Missing	2 (0.2%)	–	2 (0.2%)	–	
Diagnosis	Psychotic disorders	739 (77.3%)	5 (71.4%)	734 (77.3%)	734 (77.3%)	
	Mood disorders	217 (22.7%)	2 (28.6%)	215 (22.7%)	215 (22.7%)	
Social functioning	Poor^#^	346 (36.2%)	3 (42.9%)	343 (36.1%	*469 (49.4%)*	*260 (27.0)*
	Good to moderate^##^	350 (36.6%	–	350 (36.9%)	*480 (50.6%)*	
	Missing	260 (27.2%)	4 (57.1%)	256 (27.0%)	–	
Alcohol abuse past 6 months	Present	258 (27.0%)	2 (28.6%)	256 (27.0%)	*259 (27.3%)*	*10 (1.0*
	Absent	688 (72.0%)	5 (71.4%)	683 (72.7%)	*690 (72.7%)*	
	Missing	10 (1.0%)	–	10 (1.1%)	–	
Drug use past year	Present	248 (25.9%)	2 (28.6%)	246 (25.9%)	246 (25.9%)	11 (1.2)
	Absent	708 (74.1%)	5 (71.4%)	703 (74.1%)	703 (74.1%)	
Co-morbid PTSD	Present	184 (19.2%)	2 (28.6%)	182 (19.2%)	*185 (19.5%)*	
	Absent	761 (79.6%)	5 (71.4%)	756 (79.7%)	*764 (80.5%)*	
	Missing	11 (1.2%)	–	11 (1.2%)	–	
Childhood neglect	Present	633 (66.2%)	5 (71.4%)	628 (66.2%)	628 (66.2%)	
	Absent	323 (33.8%)	2 (28.6%)	321 (33.8%)	321 (33.8%)	
Childhood physical abuse	Present	401 (41.9%)	4 (57.1%)	397 (41.8%)	397 (41.8%)	
	Absent	555 (58.1%)	3 (42.9%)	552 (58.2%)	552 (58.2%)	
Childhood sexual abuse	Present	303 (31.7%)	2 (28.6%)	301 (31.7%)	301 (31.7%)	
	Absent	653 (68.3%)	5 (71.4%)	648 (68.3%)	648 (68.3%)	
Violent perpetration past year	Present	208 (21.8%)	1 (14.3%)	207 (21.8%)	*212 (22.3%)*	*13 (1.4)*
	Absent	735 (77.9%)	6 (85.7%)	729 (76.8%)	*737 (77.7%)*	
	Missing	13 (0.3%)	–	13 (1.4%)	–	
Dispositional anger	High^¥^	495 (51.8%)	5 (71.4%)	490 (51.6%)	*494 (52.1%)*	*10 (1.0)*
	Low^¥¥^	451 (47.2%)	2 (28.6%)	449 (47.3%)	*455 (47.9%)*	
	Missing	10 (1.0%)	–	10 (1.1%)	–	

*Italic*: Imputed values.

^#^HONOS score > 9; ^##^HONOS score ≤9; ^¥^DAR score >51; ^¥¥^DAR score ≤51.

## 3 Results

### 3.1 Sample

The full sample consisted of 956 SMI patients: 608 men (64%) and 348 women (36%). Mean (M) age was 44.7 year (Standard Deviation (SD)=10.4). The majority of respondents (61%) had Dutch ethnicity. Educational level was categorized into no/primary education (23%), basic vocational education (34%), intermediate vocational or preparatory academic education (28%), and high vocational or academic education (15%). While most patients were receiving social welfare (86%), 14% were employed (in regular and/or sheltered employment). Psychotic disorder was present in 77% of the cases. Alcohol abuse and/or drug use was present in a quarter of the respondents. The respondents’ demographic and clinical characteristics were consistent with nationwide figures for SMI patients in the Netherlands (38, 39, 68) and thus representative of our target population. Full sample, outliers, and sample characteristics before and after imputation are described in **Table 1**. The outliers were mostly men living in urban conditions. The imputed covariates did not substantially differ from the original data on any of the determinants. The results presented below were based on the imputed sample.

**Table 2** shows twelve-month prevalence of personal and property crime victimization was 19% and 28%, respectively. These participants reported a total of 612 personal crimes (M=0.65; SD 2.94) and 447 property crimes (M= 0.47; SD 1.14).

**Table 2:** Descriptive statistics for 12-month prevalences and number of incidents in the current sample after removing the identified outliers (n = 7).

	Mean	Std. deviation	Median	Range	Sum total
Property crime victimization					
Prevalence (12-month)	0.28	0.45	0	0 - 1	267
Number of incidents	0.47	1.14	0	0 - 12	447
Personal crime victimization					
Prevalence (12-month)	0.19	0.39	0	0 - 1	179
Number of incidents	0.65	2.94	0	0 - 50	612

### 3.2 Determinants of victimization prevalence and number of incidents

**Figure 1:**
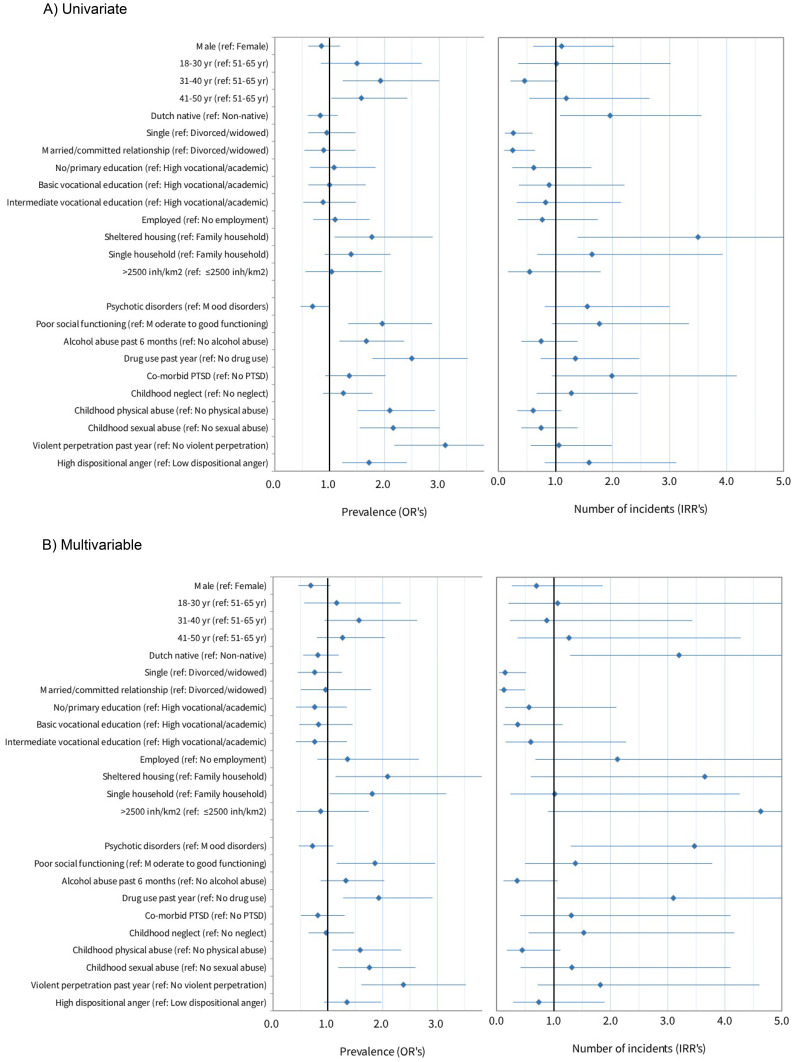
Univariable **(A)** and multivariable **(B)** determinants of personal crime victimization.

**Figure 2:**
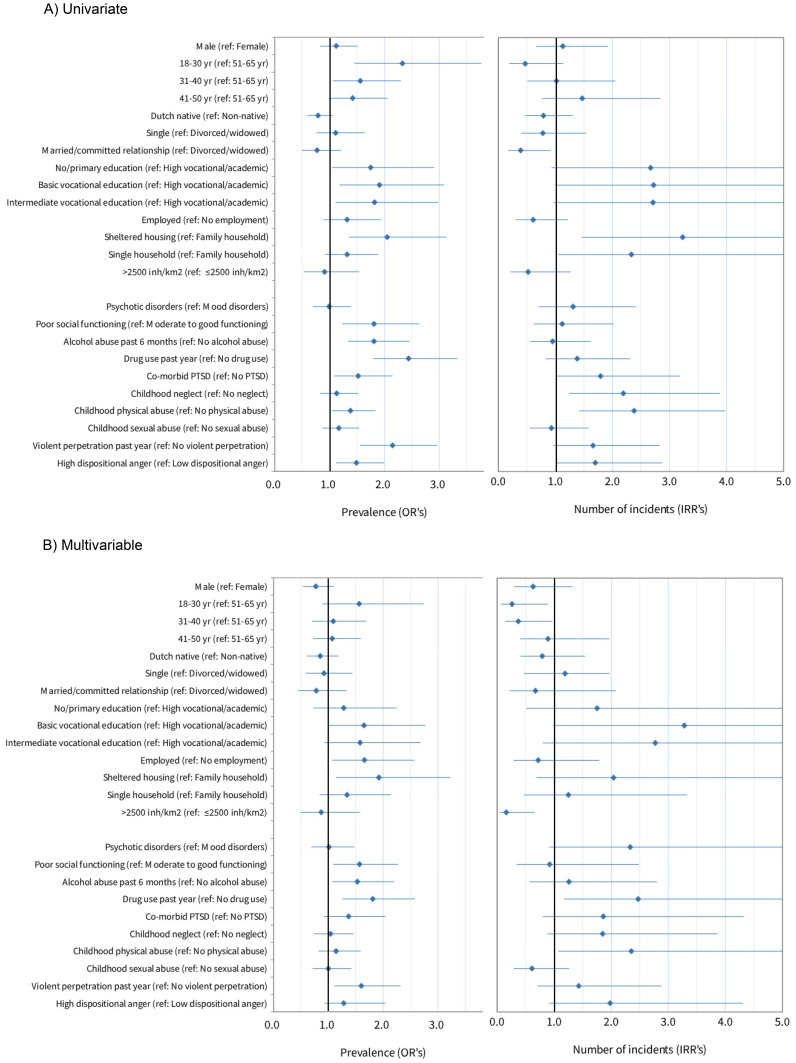
Univariable **(A)** and multivariable **(B)** determinants of property crime victimization.

**Figures 1**, **2**, and **Appendix Tables C**, **D** (**S3**, **S4**) show the determinants of personal crime victimization and property crime victimization, based on an univariable and multivariable analyses in the imputed sample from which outliers were removed. Results regarding personal and property crime are described in the following paragraphs. Results regarding total criminal victimization (personal and property crime combined) are described in **Appendix Table E** and **Appendix Figure F** (**S5**, **S6**).

#### 3.2.1 Univariable determinants of personal crime victimization prevalence

The age categories 31-40 yr. and 41-50 yr. were significantly associated with higher prevalence of personal criminal victimization compared to the oldest age category (Odds Ratio (OR)_31-40yr_: 1.95; 95%Confidence Interval (CI)_31-40yr_: 1.25-3.03; OR_41-50yr_: 1.59; 95%CI_41-50yr_: 1.04-2.44). Ethnicity, marital status, education, employment status, housing, and urbanicity did not show any significant univariable association with personal crime victimization.

Patients diagnosed with a mood disorder were more often a victim of personal crime than patients with psychotic disorders (OR: 0.68; 95%CI: 0.47-0.99). Patients with poor psychosocial functioning had a two times higher prevalence rate than patients with better levels of social functioning (OR: 2.01; 95%CI: 1.31-3.11). Both alcohol abuse (OR: 1.67; 95%CI: 1.18-2.36) and drug use (OR: 2.51; 95%CI: 1.78-3.53) were associated with higher prevalence of personal victimization. Physical and sexual abuse during childhood were also associated with over two times higher prevalence rates of personal victimization (OR_physical_: 2.11; 95%CI_physical_: 1.52-2.92; OR_sexual_: 2.17; 95%CI_sexual_: 1.56-3.03). Violent perpetration was associated with a threefold higher prevalence (OR: 3.12; 95%CI: 2.19-4.44). Finally, patients with high levels of dispositional anger showed an increased risk for personal victimization (OR: 1.72; 95%CI: 1.23-2.41). Co-morbid PTSD and childhood neglect were not significantly associated with personal crime victimization.

#### 3.2.2 Multivariable determinants of personal crime victimization prevalence

In the multivariable logistic model, and based on the sandwich estimator, all demographic and clinical determinants proved important, e.g. removal proved to change remaining coefficients to change >20%.

The impact of most univariable clinical determinants was confirmed in the final multivariable logistic model. Patients with poor psychosocial functioning were more likely to become victim of personal crime (OR: 1.72; 95%CI_S_: 1.08-2.75). Additionally, drug use remained a significant determinant for personal crime (OR: 1.95; 95%CI_S_: 1.29-2.94). Prevalence rates for victims of childhood physical abuse (OR: 1.57; 95%CI_S_: 1.08-2.28) or childhood sexual abuse (OR: 1.76; 95%CI_S_: 1.19-2.61) were increased, as well as prevalence rates for violent perpetrators (OR: 2.41; 95%CI_S_: 1.62-3.59). The patient’s diagnosis, alcohol abuse, or dispositional anger no longer showed a significant impact on personal victimization risk in the multivariable logistic model. The final multivariable model explained a moderate 25.0% of the variance of personal crime victimization.

#### 3.2.3 Univariable determinants of the frequency of personal crimes victimization

Dutch native patients did not show a higher risk of being victimized, but those who were victimized reported two times more incidents than non-native patients (Incident Rate Ratio (IRR): 1.97; 95%CI: 1.08-3.59). Divorced or widowed reported four times more incidents than patients in a committed relationship (IRR: 0.25; 95%CI: 0.10-0.64), as well as single patients (IRR: 0.26; 95%CI: 0.12-0.60). Patients victimized living in sheltered housing with other patients showed more than twofold more incidents (IRR: 2.14; 95%CI: 1.02-4.48) than patients living in single-person households. None of the other demographic or clinical determinants showed an impact on the number of personal crime incidents experienced by a victim.

#### 3.2.4 Multivariable determinants of the frequency of personal crime victimization

In the multivariable hurdle model, the impact of Dutch ethnicity on the frequency of personal crime victimization became more pronounced (IRR: 3.56; 95%CI_S_: 1.10-10.20). Similar effects were shown with regard to the decreased risk of single patients (IRR: 0.14; 95%CI_S_: 0.03-0.68) and patients in a committed relationship (IRR: 0.11; 95%CI_S_: 0.02-0.54) compared to divorced and widowed patients.

Two of the clinical determinants were found to be related to higher numbers of incidents in victims in the multivariable hurdle model. The number of incidents was more than three times higher for patients diagnosed with a psychotic disorder (IRR: 3.36; 95%CI_S_: 1.26-8.96) than for patients diagnosed with a mood disorder. Drug use was a significant determinant for personal crime frequency, showing almost four times more incidents in victims that used drugs (IRR: 3.85; 95%CI: 1.07-13.88).

### 3.3 Property crime victimization

#### 3.3.1 Univariable determinants of property crime victimization prevalence

Of the socio-demographic characteristics, younger age categories were significantly associated with higher prevalence of property criminal victimization compared to the oldest age category (OR_18-30yr_: 2.33; 95%CI_18-30yr_: 1.45-3.77; OR_31-40yr_: 1.56; 95%CI_31-40yr_: 1.06-2.30). Property crime victimization was almost two times more common among patients with low educational levels than among those with high vocational or academic educational levels (OR: 0.57; 95%CI: 0.34-0.96). Patients living in sheltered housing showed a more than 1.5-fold higher prevalence (OR: 1.56; 95%CI: 1.09-2.23) than patients living in a single household. Gender, ethnicity, marital status, employment status, and urbanicity did not show any univariable significant association with the prevalence of property crime victimization.

Patients with poor psychosocial functioning had almost two times higher prevalence rates than patients with better levels of social functioning (OR: 1.881; 95%CI: 1.28-2.74). Alcohol abuse (OR: 1.81; 95%CI: 1.33-2.47) and drug use (OR: 2.44; 95%CI: 1.80-3.33) were associated with a doubling of the risk of property crime victimization. Patients with co-morbid PTSD also reported more often to be a victim of property crime (OR: 1.52: 95%CI: 1.07-2.15). Patients who had experienced physical abuse during childhood were also more likely to become victim of property crime (OR: 1.38; 95%CI: 1.04-1.84). Violent perpetration was associated with a twofold higher prevalence (OR: 2.15; 95%CI: 1.56-2.98). Finally, patients with high levels of dispositional anger showed a higher risk for property victimization (OR: 1.50; 95%CI: 1.12-2.00). The patient’s diagnosis, childhood neglect and childhood sexual abuse were not significantly associated with the prevalence of property crime victimization.

#### 3.3.2 Multivariable determinants of property crime prevalence

In the multivariable logistic model, all demographic and clinical factors were kept in the model. Either they contributed to the overall fit of the model or their removal resulted in a >20% change of the coefficients of the main determinants. Gender, age, ethnicity, marital status, educational level, housing and urbanicity did not show significant associations with property crime prevalence in the full logistic model. Only employment status was significantly related to an increased risk of property crime victimization in the full logistic model. Patients with a paid job had a higher risk of property crime victimization (OR: 1.64; 95%CI_s_: 1.06-2.53).

With respect to the clinical determinants, co-morbid PTSD, childhood neglect, childhood physical abuse, and dispositional anger were no longer associated with property crime prevalence in the multivariable logistic model. Patients with poor psychosocial functioning had a higher risk for property crime victimization (OR: 1.57; 95%CI_s_:1.03-2.38). Additionally, patients with alcohol abuse (OR: 1.54; 95%CI_s_: 1.07-2.24) and drug use (OR: 1.79; 95%CI_s_: 1.25-2.56) showed higher risks for property crime victimization. Finally, patients who had committed a violent crime over the past year, also had a higher risk to fall victim to a property crime (OR: 1.66; 95% CI_s_: 1.15-2.39).

#### 3.3.3 Univariable determinants of the frequency of property crime victimization

The number of property crime incidents for those who had fallen/were victim, was significantly higher for divorced or widowed patients than for lower for married patients (IRR: 0.39; 95%CI: 0.17-0.62). In line with that finding patients living in a single household reported more incidents (IRR: 0.43; 95%CI: 0.20-0.95), than patients living in a family household. None, of the other demographic characteristics were found to be related to the frequency rate of property crime in the univariable analysis.

Patients with co-morbid PTSD, survivors of childhood neglect or childhood physical abuse reported approximately twice the number of property victimization incidents (IRR_ptsd_: 1.79; 95%CI_ptsd_: 1.01-3.19; IRR_neglect_: 2.19; 95%CI_neglect_: 1.24-3.88; IRR_physical abuse_: 2.38; 95%CI_physical abuse_: 1.42-1.98. Additionally, victims of property crime that showed high levels of dispositional anger, experienced more incidents (IRR: 1.70; 95%CI: 1.01-2.87).

#### 3.3.4 Multivariable determinants of the frequency of property crime victimization

In the multivariable hurdle model and based on sandwich estimation, none of the individual demographic or clinical characteristics were significantly associated with the number of incidents a victim experienced property crime. The variance explained by the final model was a moderate 27.9% as measured by Nagelkerke’s pseudo-R^2^.

By means of sensitivity analyses, all property crime models were refitted with car-related property crime included. Results show a more pronounced impact of poor social functioning and drug use and less pronounced impact of employment (Δ β > 5%), while the impact of alcohol abuse and violent perpetration remained unaffected by the inclusion this type of property crime (see **Appendix Table G (S7)**).

## 4 Discussion

We found high prevalence of personal and property victimization in outpatients with severe mental illness, as well as several predictive determinants.

Determinants differed across crime categories, suggesting that pathways to victimization differ for personal and property crime incidents. For personal crime victimization, we found a profound impact, often both on prevalence and frequency rate of clinical determinants and childhood trauma. Higher frequency rates were found for victims with psychotic disorder. A higher prevalence risk was found among those with lower levels of psychosocial functioning and current alcohol abuse, defined as occasional excessive drinking, or drug use. In case of drug use, the number of incidents of personal crime victimization was four times higher, while alcohol abuse was related to an almost two times higher risk for this type of victimization. The risk of being personally victimized was also higher for those with a history of childhood physical and sexual trauma. Perpetrators of violence were at double odds of becoming a personal crime victim and having twice as many incidents than other non-perpetrator victims. Dutch and divorced victims experienced three to four times more incidents than non-native, married or single victims. The models explained a moderate amount of variance in crime victimization.

Turning to property crime, becoming a victim of this category was more common among younger patients, and patients with paid employment. In contrast to single-person households, patients living with others in sheltered housing had a higher risk of becoming victims of property crime. Elevated prevalence of property victimization was also associated with lower levels of social functioning, alcohol abuse, and drug use. Finally, violent perpetration indicated an increased risk of becoming a victim. Victims of childhood physical abuse were equally vulnerable to become a victim of property crime as patients without these childhood experiences. However, the number of property crime incidents reported by these victims was doubled. Although the frequencies were elevated for a variety of clinical factors, in a multivariable hurdle model most of these determinants lost their statistical significance.

Previous studies found increased victimization risk for women in the general population and female patients (2, 35, 69). However, these previous studies often estimated associations with gender using univariable models (70, 71). In the current study we did not find women to be at an increased risk for personal or property crime. A gender difference emerged only when looking at a combined category of total crime. The gender effect therefore was not a particularly robust one in the present analysis. Additionally, it has been previously reported that among SMI patients gender differences related to victimization are less profound, and it has even been suggested that the presence of SMI impacts men more than women (36). The increased vulnerability of divorced patients was shown in earlier studies as well (21, 23, 28). Our results showed that this vulnerability relates to both the number of patients being victimized, as well as the number of incidents experienced. We suggest that in many of these cases victimization could take place during or in the aftermath of the divorce, or in the context of co-parenting (72).

Living in sheltered housing was found to be a potential risk factor especially for property crime victimization, impacting both prevalence and frequency rates. This is particularly poignant since sheltered living is supposed to protect and facilitate recovery of vulnerable patients (73). The elevated risk of shared sheltered housing, in contrast to single housing, remained significant after adjusting for socio-demographic and a variety of clinical characteristics, suggesting that mechanisms unique to those housing arrangements pose an independent risk for its inhabitants. The downsides of sheltered housing have been described before, however often in the context of qualitative studies (73–76).

We found consistent and strong associations between violent perpetration and victimization. This interrelationship has been documented previously (1, 21, 35, 77), and so has been the impact of childhood trauma (22, 78). For both personal as well as property crime victimization, we found a strong and consistent association with an overall lower level of functioning (more symptoms, more impairments, problems with substance use) in victims. In the multivariable hurdle model these factors, which suggest a heightened level of psychopathology, remained relevant. However, when including a more robust form of parameter estimation, the coefficients lost significance in favor to the factors indicating a risk-taking lifestyle.

Although documented previously, and strongly connected with the cycle of violence paradigm, we did not find co-morbid PTSD diagnosis to be an important determinant for personal crime victimization (22, 67, 75, 79). We speculate that within the subgroup of SMI patients, PTSD symptomatology is closely interwoven with the overall level of psychopathology and therefore cannot show the same discriminant value it has in less affected samples.

### 4.1 Strengths and limitations

The most prominent strengths of this study were its large sample size, random sampling method and high response rate offering a unique perspective on victimization based on an hard to reach SMI population in Europe. The sample size allowed us to reliably estimate small to medium sized associations with personal and property crime victimization in the multivariable models. Furthermore, the representative characteristics of our sample offer a strong case for the current results to be generalized on a population level beyond the sample of SMI patients reported here.

We also note several limitations of the current study. Generally, falling victim to crime, especially falling victim to more severe forms of crime over a 12-month period is considered a rare event. Rare event models are prone to speculative results and over-interpretation. We used hurdle regression models to analyze our rare and overdispersed data. Furthermore, we chose to combine our crime incidents into two categories. In doing so, we found robust determinants of personal and property crime victimization prevalence. However, by modelling categories, we lost the ability to distinguish unique determinants for unique crime incidents and we might have lost power to detect more modest associations (80). This might have limited the usefulness of our models with regard to more rare crime incidents, such as sexual assault. The estimations of the determinants of the number of victimization incidents, were less robust. We speculate that this results from the existence of specific (high risk) subpopulations within our sample (34).

Another limitation concerns the use of self-reported data, which is vulnerable to bias. Patients might have over-reported or under-reported their symptoms and use of drugs and alcohol due to unmeasured variables. It is unknown how these divergent possibilities would affect the magnitude and directions of the association reported here. Crime victimization numbers were also based on self-report. However, the IVM questionnaire is developed specifically to avoid recall bias and telescoping (i.e., the tendency to report impactful events closer in more recent history than is true) and considered the golden standard in Dutch crime research (40). Finally, the data was collected between 2010 and 2012, which might have impacted the generalizability of the prevalence and incidence rates, but most likely does not impact the underlying dynamics causing SMI patients to become victims of crime.

### 4.2 Conclusions and clinical implications

We found high annual prevalence rates of personal and property victimization of 19% and 28% respectively. For property crime, we found the strongest determinants in the clinical category, in particular substance abuse and poor social functioning. However, we also found predictive associations among the victimological determinants (i.e. being a perpetrator of violence) and the sociodemographic determinants (i.e. being unemployed). For personal crime, clinical determinants, consisting of drug use and poor social, functioning again emerged as the strongest predictors. In the victimological category, perpetration of violence, but also childhood abuse were predictive of personal crime.

This study provides more evidence for clinicians to become more aware of criminal victimization as well as perpetration, since both are prevalent and strongly interrelated. This perpetuation of violence, from childhood into adulthood and between victimization and perpetration incidents, is often described and is referred to as the cycle of violence (79). To break this cycle of violence, we stress the importance of early intervention, starting with the prevention of childhood neglect and abuse, especially because these factors also have an important role in developing adult psychopathology. We speculate that the pathways to personal victimization (e.g., physical and sexual threats and violent acts) are determined more profoundly by this cycle of violence than property victimization (e.g., theft, vandalism) which seems to be impacted more by a risk-taking lifestyle and opportunity (e.g., the presence of valuables). Intervention studies reported that clinicians feel inhibited to discuss these topics with their patients and suggested that less than 10% of the patients are detected as victims by the primary clinician (81–83). Training of clinicians, mandatory screening or AI-supported screening might help overcome the hesitation surrounding crime victimization (84–86).

Considering the high prevalence and frequency, there is a strong need for evidence-based interventions to prevent victimization. Interventions should target specific risk profiles. We distinguished specific risk profiles for personal and property crimes, patients with situational or chronic lifetime risk patterns, risk profiles related to failing coping strategies, as well as profiles related to risk taking and disorganized behavior (87). Once SMI patients have been victimized trauma-focused therapy is safe and effectively reduces effects on psychiatric symptoms and improves social functioning (88, 89). Given the impact of the cycle of violence, we urge early intervention as well as a nuanced perspective on the interrelationship of perpetration and victimhood. We further suggest the staff of sheltered housing to remain alert for crime incidents, monitoring both co-habitants as well as contact from the wider community. Theft and vandalism could be prevented by providing personal lockers or helping inhabitants make sure their doors remain locked.

To conclude, this study underscores the vulnerable position of SMI patients. The high prevalence of crime victimization and the strong interrelationship with violent perpetration among outpatients urges clinicians to engage their patients in discussing the impact of crime and violence in their lives. Overall, the clinical determinants were most important, but the interplay with other demographic and victimological factors stress the importance of a holistic perspective on victimization.

## Data Availability

The data is not available due to the sensitive nature of the population under study. Requests to access the datasets should be directed to a.kamperman@erasmusmc.nl.

## Footnotes

1 Carroll RJ, Wang S, Simpson DG, Stromberg AJ, Ruppert D. The sandwich (robust covariance matrix) estimator. (Unpublished manuscript)
