# Supplementary material for: Determinants of victimization in patients with severe mental illness: results from a nation-wide cross-sectional survey in the Netherlands
Source: Front Psychiatry. 2025 Mar 17;16:1511841. doi: 10.3389/fpsyt.2025.1511841 (PMC11955743; doi:10.3389/fpsyt.2025.1511841)
Supplement: Supplementary file 1 [file DataSheet1.zip › Appendix Table B.DOCX]

Appendix Table B: Determinants of personal crime victimization^a^ estimated on the imputed dataset from which outliers were removed (N=949): Results from univariable regression analyses, results from stepwise backward multivariable hurdle regression analyses, and final model with re-estimated standard errors using sandwich estimation.

|  |  | Univariable models | | Final multivariable model | | Final multivariable model, including sandwich estimator | |
| --- | --- | --- | --- | --- | --- | --- | --- |
|  |  | Binomial logit  Prevalence | Negative binomial count  Number of incidents | Binomial logit  Prevalence | Negative binomial count  Number of incidents | Binomial logit  Prevalence | Negative binomial count  Number of incidents |
|  |  | OR (95%CI) | IRR (95%CI) | OR (95%CI) | IRR (95%CI) | OR (95%CI_S_) | IRR (95%CI_S_) |
| Sex | Male | 0.85 (0.61-1.19) | 1.12 (0.61-2.05) | 0.72 (0.48-1.08) | 0.74 (0.29-1.88) | 0.72 (0.47-1.09) | 0.74 (0.27-1.99) |
|  | Female | 1 | 1 | 1 | 1 | 1 | 1 |
| Age | 18–30 yr. | 1.53 (0.85-2.75) | 1.02 (0.35-3.00) | 1.16 (0.58-2.32) | 1.17 (0.23-5.95) | 1.16 (0.58-2.32) | 1.17 (0.22-6.26) |
|  | 31-40 yr. | 1.95 (1.25-3.03)** | 0.46 (0.21-1.03) | 1.59 (0.95-2.65) | 0.82 (0.21-3.26) | 1.59 (0.94-2.67) | 0.82 (0.20-3.49) |
|  | 41-50 yr. | 1.59 (1.04-2.44)* | 1.19 (0.54-2.63) | 1.29 (0.80-2.07) | 1.24 (0.36-4.28) | 1.29 (0.80-2.07) | 1.24 (0.36-4.32) |
|  | 51-65 yr. | 1 | 1 | 1 | 1 | 1 | 1 |
| Ethnicity | Dutch native | 0.83 (0.59-1.15) | 1.97 (1.08-3.59)* | 0.82 (0.56-1.21) | 3.36 (1.35-8.40)** | 0.82 (0.55-1.21) | 3.36 (1.10-10.20)* |
|  | Non-native | 1 | 1 | 1 | 1 | 1 | 1 |
| Marital status | Single | 0.94 (0.61-1.46) | 0.26 (0.12-0.60)** | 0.75 (0.45-1.26) | 0.14 (0.04-0.50)** | 0.75 (0.45-1.27) | 0.14 (0.03-0.68)* |
|  | Married/ committed relationship | 0.89 (0.54-1.47) | 0.25 (0.10-0.64)** | 0.94 (0.51-1.73) | 0.11 (0.03-0.43)** | 0.94 (0.50-1.75) | 0.11 (0.02-0.54)** |
|  | Divorced/ widowed | 1 | 1 | 1 | 1 | 1 | 1 |
| Education | Low | 1 | 1 | 1 | 1 | 1 | 1 |
|  | Mid-Low | 0.94 (0.61-1.45) | 1.39 (0.64-3.05) | 1.10 (0.68-1.77) | 0.75 (0.27-2.13) | 1.10 (0.68-1.79) | 0.75 (0.27-2.08) |
|  | Mid-High | 0.82 (0.52-1.30) | 1.29 (0.56-2.99) | 1.03 (0.61-1.72) | 1.27 (0.33-4.86) | 1.03 (0.60-1.75) | 1.27 (0.31-5.16) |
|  | High | 0.93 (0.55-1.59) | 1.57 (0.60-4.11) | 1.32 (0.72-2.44) | 1.85 (0.52-6.56) | 1.32 (0.71-2.47) | 1.85 (0.53-6.52) |
| Employment | Yes | 1.11 (0.71-1.74) | 0.54 (0.34-1.72) | 1.32 (0.79-2.20) | 1.89 (0.61-5.87) | 1.32 (0.80-2.16) | 1.89 (0.72-4.96) |
|  | No | 1 | 1 | 1 | 1 | 1 | 1 |
| Housing | Sheltered housing | 1.28 (0.86-1.92) | 2.14 (1.02-4.48)* | 1.12 (0.70-1.78) | 2.72 (0.80-9.20) | 1.12 (0.70-1.79) | 2.72 (0.71-10.45) |
|  | Family household | 0.73 (0.48-1.01) | 0.61 (0.26-1.46) | 0.58 (0.34-0.99)* | 1.21 (0.32-4.60) | 0.58 (0.33-1.01) | 1.21 (0.32-4.50) |
|  | Single household | 1 | 1 | 1 | 1 | 1 | 1 |
| Urbanity | > 2500 inh./km2 | 1.04 (0.56-1.95) | 0.55 (0.17-1.78) | 0.88 (0.44-1.75) | 4.38 (0.87-22.03) | 0.88 (0.42-1.82) | 4.38 (0.54-35.73) |
|  | ≤ 2500 inh./km2 | 1 | 1 | 1 | 1 | 1 | 1 |
| Diagnosis | Psychotic disorders | 0.68 (0.47-0.99)* | 1.57 (0.82-3.02) | 0.73 (0.48-1.11) | 3.36 (1.29-8.76)* | 0.73 (0.48-1.11) | 3.36 (1.26-8.96)* |
|  | Mood disorders | 1 | 1 | 1 | 1 | 1 | 1 |
| Social functioning | Poor^#^ | 2.01 (1.31-3.11)** | 1.72 (0.92-3.24) | 1.72 (1.07-2.77)* | 1.07 (0.39-2.91) | 1.72 (1.08-2.75)* | 1.07 (0.41-2.75) |
|  | Moderate to good^##^ | 1 | 1 | 1 | 1 | 1 | 1 |
| Alcohol abuse  past 6 months | Present | 1.67 (1.18-2.36)** | 0.75 (0.40-1.38) | 1.32 (0.87-2.00) | 0.32 (0.12-0.90)* | 1.32 (0.86-2.03) | 0.32 (0.09-1.20) |
|  | Absent | 1 | 1 | 1 | 1 | 1 | 1 |
| Drug use past year | Present | 2.51 (1.78-3.53)*** | 1.34 (0.73-2.45) | 1.95 (1.29-2.94)** | 3.85 (1.37-10.87)* | 1.95 (1.29-2.94)** | 3.85 (1.07-13.88)* |
|  | Absent | 1 | 1 | 1 | 1 | 1 | 1 |
| Co-morbid PTSD | Present | 1.34 (0.91-1.98) | 1.93 (0.94-3.93) | 0.80 (0.50-1.28) | 1.46 (0.45-4.69) | 0.80 (0.51-1.27) | 1.46 (0.48-4.44) |
|  | Absent | 1 | 1 | 1 | 1 | 1 | 1 |
| Childhood neglect | Present | 1.25 (0.88-1.78) | 1.26 (0.66-2.40) | 0.96 (0.64-1.44) | 1.72 (0.64-4.64) | 0.96 (0.65-1.44) | 1.72 (0.68-4.38) |
|  | Absent | 1 | 1 | 1 | 1 | 1 | 1 |
| Childhood physical abuse | Present | 2.11 (1.52-2.94)*** | 0.60 (0.33-1.09) | 1.57 (1.07-2.31)* | 0.43 (0.17-1.06) | 1.57 (1.08-2.28)* | 0.43 (0.18-1.03) |
|  | Absent | 1 | 1 | 1 | 1 | 1 | 1 |
| Childhood sexual abuse | Present | 2.17 (1.56-3.03)*** | 0.75 (0.41-1.35) | 1.76 (1.20-2.60)** | 1.31 (0.53-3.23) | 1.76 (1.19-2.61)** | 1.31 (0.55-3.08) |
|  | Absent | 1 | 1 | 1 | 1 | 1 | 1 |
| Violent perpetration past year | Present | 3.12 (2.19-4.44)*** | 1.07 (0.57-2.00) | 2.41 (1.62-3.58)*** | 2.02 (0.79-5.13) | 2.41 (1.62-3.59)*** | 2.02 (0.80-5.10) |
|  | Absent | 1 | 1 | 1 | 1 | 1 | 1 |
| Dispositional anger | High^¥^ | 1.72 (1.34-2.41)** | 1.59 (0.81-3.13) | 1.37 (0.94-2.00) | 0.72 (0.28-1.87) | 1.37 (0.95-1.99) | 0.72 (0.23-2.24) |
|  | Low^¥¥^ | 1 | 1 | 1 | 1 | 1 | 1 |
| * p<0.05 ;** p<0.01; *** p<0.001  ^#^ HONOS score > 9; ^##^  HONOS score =< 9  ^¥^ DAR score >51; ^¥¥^ DAR score <=51  ^a^ Comprises sexual harassment or assault, threatened with violence, and physical assault  Sex, urbanity and social functioning are included in the multivariable model irrespective of model building criteria (grey shading) | | | | | | | |
