# Supplementary material for: Determinants of victimization in patients with severe mental illness: results from a nation-wide cross-sectional survey in the Netherlands
Source: Front Psychiatry. 2025 Mar 17;16:1511841. doi: 10.3389/fpsyt.2025.1511841 (PMC11955743; doi:10.3389/fpsyt.2025.1511841)
Supplement: Supplementary file 1 [file DataSheet1.zip › Appendix Table C.DOCX]

Appendix Table C: Determinants of property crime victimization^a^ estimated on the imputed dataset from which outliers were removed (N=949): Results from univariable regression analyses, results from stepwise backward multivariable hurdle regression analyses, and final model with re-estimated standard errors using sandwich estimation.

|  |  | Univariable models | | Final multivariable model | | Final multivariable model, including sandwich estimator | |
| --- | --- | --- | --- | --- | --- | --- | --- |
|  |  | Binomial logit  Prevalence | Negative binomial count  Number of incidents | Binomial logit  Prevalence | Negative binomial count  Number of incidents | Binomial logit  Prevalence | Negative binomial count  Number of incidents |
|  |  | OR (95%CI) | IRR (95%CI) | OR (95%CI) | IRR (95%CI) | OR (95%CI_S_) | IRR (95%CI_S_) |
| Sex | Male | 1.12 (0.83-1.51) | 1.13 (0.66-1.92) | 0.78 (0.55-1.12) | 0.62 (0.30-1.29) | 0.78 (0.54-1.12) | 0.62 (0.23-1.67) |
|  | Female | 1 | 1 | 1 | 1 | 1 | 1 |
| Age | 18–30 yr. | 2.33 (1.45-3.77)** | 0.47 (0.19-1.14) | 1.55 (0.88-2.71) | 0.26 (0.08-0.90)* | 1.55 (0.88-2.72) | 0.26 (0.04-1.82) |
|  | 31-40 yr. | 1.56 (1.06-2.31)* | 1.02 (0.51-2.05) | 1.08 (0.70-1.69) | 0.37 (0.14-0.95) | 1.08 (0.69-1.69) | 0.37 (0.05-3.03) |
|  | 41-50 yr. | 1.42 (0.99-2.06) | 1.47 (0.76-2.84) | 1.07 (0.72-1.59) | 0.91 (0.41-2.00) | 1.07 (0.72-1.59) | 0.91 (0.35-2.38) |
|  | 51-65 yr. | 1 | 1 | 1 | 1 | 1 | 1 |
| Ethnicity | Dutch native | 0.79 (0.59-1.06) | 0.79 (0.47-1.31) | 0.85 (0.61-1.19) | 0.78 (0.40-1.52) | 0.85 (0.61-1.20) | 0.78 (0.43-1.41) |
|  | Non-native | 1 | 1 | 1 | 1 | 1 | 1 |
| Marital status | Single | 1.11 (0.76-1.64) | 0.79 (0.40-1.54) | 0.91 (0.59-1.42) | 1.21 (0.49-3.02) | 0.91 (0.59-1.42) | 1.21 (0.53-2.78) |
|  | Married/ committed relationship | 0.77 (0.49-1.21) | 0.39 (0.17-0.62)* | 0.77 (0.45-1.32) | 0.65 (0.21-1.98) | 0.77 (0.45-1.32) | 0.65 (0.25-1.69) |
|  | Divorced/ widowed | 1 | 1 | 1 | 1 | 1 | 1 |
| Education | Low | 1 | 1 | 1 | 1 | 1 | 1 |
|  | Mid-Low | 1.09 (0.75-1.60) | 1.02 (0.52-1.97) | 1.29 (0.86-1.95) | 1.89 (0.83-4.29) | 1.29 (0.85-1.96) | 1.89 (0.32-11.07) |
|  | Mid-High | 1.04 (0.70-1.55) | 1.02 (0.51-2.03) | 1.26 (0.82-1.95) | 1.62 (0.66-3.99) | 1.26 (0.81-1.96) | 1.62 (0.47-5.61) |
|  | High | 0.57 (0.34-0.96)* | 0.37 (0.13-1.08) | 0.78 (0.44-1.37) | 0.58 (0.17-1.97) | 0.78 (0.44-1.38) | 0.58 (0.18-1.83) |
| Employment | Yes | 1.32 (0.89-1.94) | 0.61 (0.30-1.22) | 1.64 (1.05-2.54)* | 0.70 (0.29-1.71) | 1.64 (1.06-2.53)* | 0.70 (0.23-2.10) |
|  | No | 1 | 1 | 1 | 1 | 1 | 1 |
| Housing | Sheltered housing | 1.56 (1.09-2.23)* | 1.14 (0.71-2.74) | 1.41 (0.94-2.10) | 1.50 (0.68-3.29) | 1.41 (0.94-2.12) | 1.50 (0.43-5.29) |
|  | Family household | 0.76 (0.53-1.10) | 0.43 (0.20-0.95)* | 0.75 (0.48-1.19) | 0.84 (0.32-2.24) | 0.75 (0.47-1.20) | 0.84 (0.30-2.38) |
|  | Single household | 1 | 1 | 1 | 1 | 1 | 1 |
| Urbanity | > 2500 inh./km2 | 0.91 (0.53-1.54) | 0.52 (0.21-1.27) | 0.88 (0.49-1.59) | 0.16 (0.04-0.64)** | 0.88 (0.49-1.58) | 0.16 (0.00-28.73) |
|  | ≤ 2500 inh./km2 | 1 | 1 | 1 | 1 | 1 | 1 |
| Diagnosis | Psychotic disorders | 0.99 (0.70-1.39) | 1.31 (0.71-2.41) | 1.02 (0.70-1.49) | 2.33 (0.93-5.82) | 1.02 (0.70-1.49) | 2.33 (0.35-15.43) |
|  | Mood disorders | 1 | 1 | 1 | 1 | 1 | 1 |
| Social functioning | Poor^#^ | 1.88 (1.28-2.74)** | 1.21 (0.61-2.41) | 1.57 (1.03-2.38)* | 0.88 (0.41-1.88) | 1.57 (1.03-2.38)* | 0.88 (0.34-2.23) |
|  | Moderate to good^##^ | 1 | 1 | 1 | 1 | 1 | 1 |
| Alcohol abuse  past 6 months | Present | 1.81 (1.33-2.47)*** | 0.95 (0.56-1.61) | 1.55 (1.07-2.23)* | 1.26 (0.57-2.79) | 1.54 (1.07-2.24)* | 1.26 (0.52-3.04) |
|  | Absent | 1 | 1 | 1 | 1 | 1 | 1 |
| Drug use past year | Present | 2.44 (1.80-3.33)*** | 1.38 (0.83-2.31) | 1.79 (1.25-2.57)** | 2.51 (1.20-5.28)* | 1.79 (1.25-2.56)** | 2.51 (0.32-19.47) |
|  | Absent | 1 | 1 | 1 | 1 | 1 | 1 |
| Co-morbid PTSD | Present | 1.52 (1.07-2.15)* | 1.79 (1.01-3.19)* | 1.36 (0.91-2.03) | 1.81 (0.79-4.14) | 1.36 (0.91-2.02) | 1.81 (0.39-8.38) |
|  | Absent | 1 | 1 | 1 | 1 | 1 | 1 |
| Childhood neglect | Present | 1.13 (0.83-1.52) | 2.19 (1.24-3.88)** | 1.03 (0.73-1.44) | 1.86 (0.89-3.87) | 1.02 (0.73-1.45) | 1.86 (0.71-4.89) |
|  | Absent | 1 | 1 | 1 | 1 | 1 | 1 |
| Childhood physical abuse | Present | 1.38 (1.04-1.84)* | 2.38 (1.42-3.98)** | 1.13 (0.81-1.58) | 2.34 (1.07-5.10) | 1.13 (0.82-1.56) | 2.34 (1.01-5.40) |
|  | Absent | 1 | 1 | 1 | 1 | 1 | 1 |
| Childhood sexual abuse | Present | 1.17 (0.87-1.58) | 0.93 (0.55-1.58) | 1.00 (0.70-1.42) | 0.60 (0.29-1.25) | 1.00 (0.71-1.41) | 0.60 (0.30-1.20) |
|  | Absent | 1 | 1 | 1 | 1 | 1 | 1 |
| Violent perpetration past year | Present | 2.15 (1.56-2.98)*** | 1.65 (0.96-2.81) | 1.66 (1.16-2.39)** | 1.44 (0.72-2.87) | 1.66 (1.15-2.39)** | 1.43 (0.67-3.09) |
|  | Absent | 1 | 1 | 1 | 1 | 1 | 1 |
| Dispositional anger | High^¥^ | 1.50 (1.12-2.00)** | 1.70 (1.01-2.87)* | 1.29 (0.93-1.78) | 1.97 (0.92-4.22) | 1.29 (0.93-1.77) | 1.97 (0.38-10.32) |
|  | Low^¥¥^ | 1 | 1 | 1 | 1 | 1 | 1 |
| * p<0.05 ;** p<0.01; *** p<0.001  ^#^ HONOS score > 9; ^##^  HONOS score =< 9  ^¥^ DAR score >51; ^¥¥^ DAR score <=51  ^a^ Comprises burglary, burglary attempt, bike theft, pickpocketing, robbery, theft (other)  Sex, urbanity and social functioning are included in the multivariable model irrespective of model building criteria (grey shading) | | | | | | | |
