# Supplementary material for: Determinants of victimization in patients with severe mental illness: results from a nation-wide cross-sectional survey in the Netherlands
Source: Front Psychiatry. 2025 Mar 17;16:1511841. doi: 10.3389/fpsyt.2025.1511841 (PMC11955743; doi:10.3389/fpsyt.2025.1511841)
Supplement: Supplementary file 1 [file DataSheet1.zip › Appendix Figure D.DOCX]

Appendix Figure D: Univariable and multivariable determinants of crime victimization

Univariable determinants


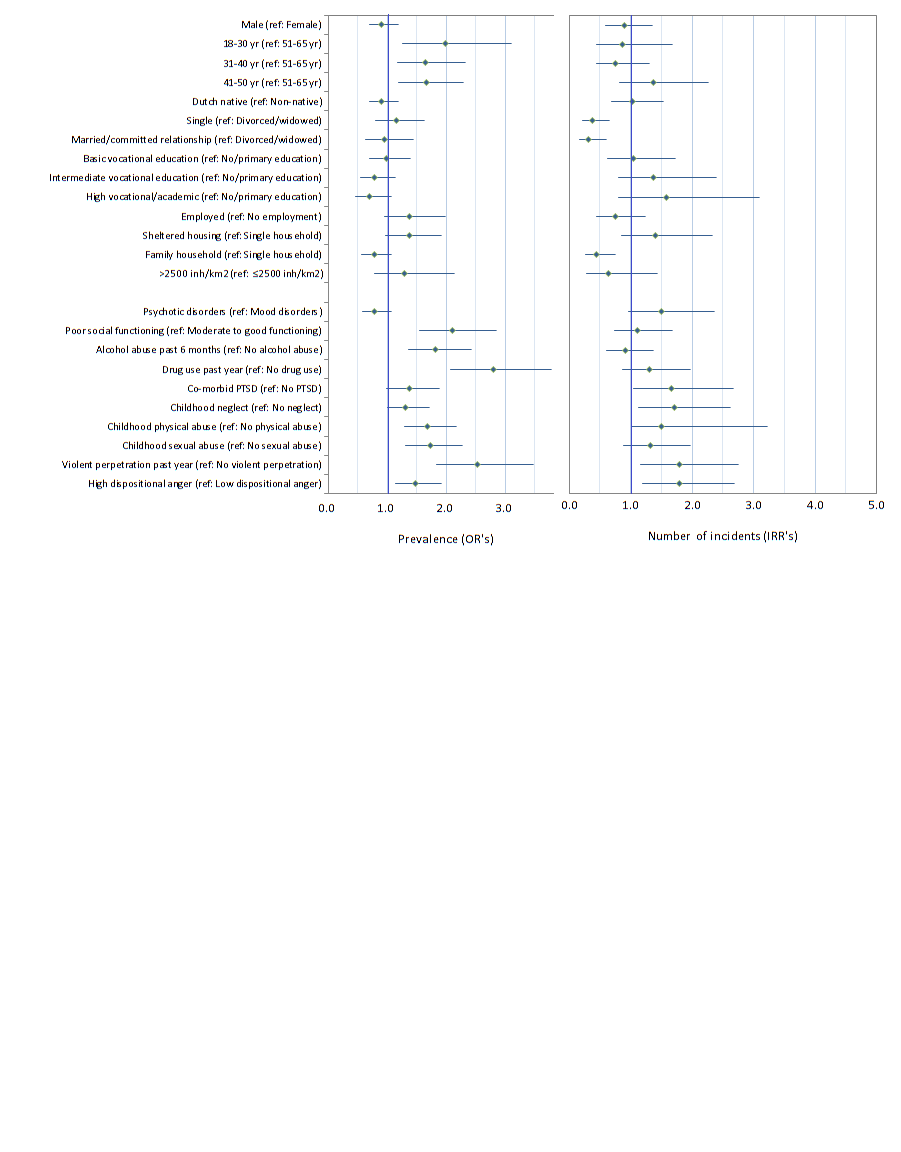


Multivariable determinants


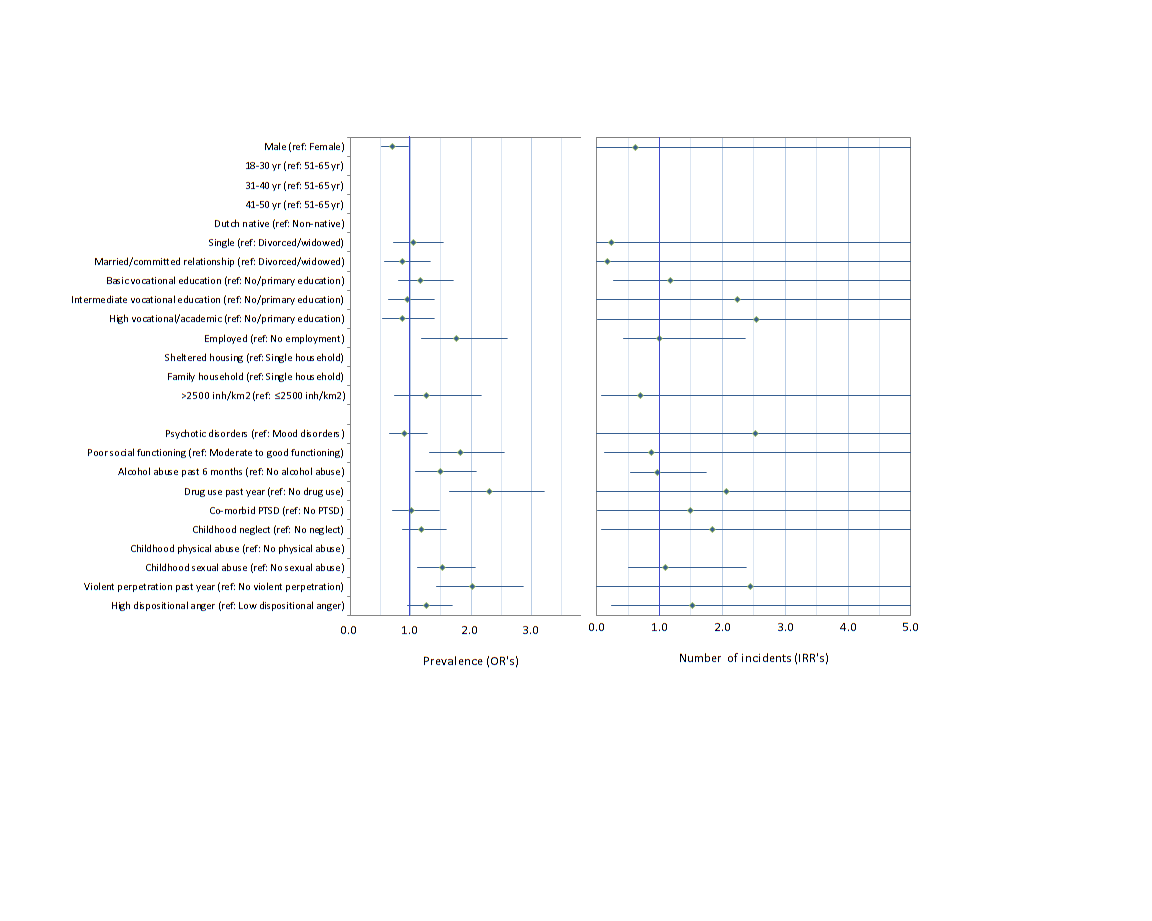


Note: Results obtained from negative binomial hurdle models on crime victimization (all types), N=949 (imputed dataset from which outliers were removed). Odds ratio’s (OR) with 95% confidence interval: probabilities of experiencing one or more crime victimization incidents; incident rate ratio’s (IRR) with 95% confidence interval: number of crime victimization incidents. Standard errors are calculated using sandwich estimation. Confidence intervals are not plotted beyond OR of 4.0 or IRR of 5.0. Multivariable results obtained using a stepwise backward selection process.
