# Supplementary material for: Determinants of victimization in patients with severe mental illness: results from a nation-wide cross-sectional survey in the Netherlands
Source: Front Psychiatry. 2025 Mar 17;16:1511841. doi: 10.3389/fpsyt.2025.1511841 (PMC11955743; doi:10.3389/fpsyt.2025.1511841)
Supplement: Supplementary file 1 [file DataSheet1.zip › Appendix Table E.DOCX]

Appendix Table E: Determinants of property crime victimization, including car related crimes^a^ estimated on the imputed dataset from which outliers were removed (N=949): Results from univariable regression analyses, results from stepwise backward multivariable hurdle regression analyses, and final model with re-estimated standard errors using sandwich estimation.

|  |  | Univariable models | | Final multivariable model | | Final multivariable model, including sandwich estimator | |
| --- | --- | --- | --- | --- | --- | --- | --- |
|  |  | Binomial logit  Prevalence | Negative binomial count  Number of incidents | Binomial logit  Prevalence | Negative binomial count  Number of incidents | Binomial logit  Prevalence | Negative binomial count  Number of incidents |
|  |  | OR (95%CI) | IRR (95%CI) | OR (95%CI) | IRR (95%CI) | OR (95%CI_S_) | IRR (95%CI_S_) |
| Sex | Male | 1.16 (0.87-1.57) | 1.09 (0.64-1.85) | 0.82 (0.58-1.16) | 0.71 (0.35-1.42) | 0.82 (0.57-1.16) | 0.71 (0.25-1.98) |
|  | Female | 1 | 1 | 1 | 1 | 1 | 1 |
| Age | 18–30 yr. | 2.49 (1.54-4.00)** | 0.45 (0.19-1.09) | 1.65 (0.96-2.83) | 0.28 (0.09-0.92)* | 1.65 (0.96-2.83) | 0.28 (0.06-1.37) |
|  | 31-40 yr. | 1.57 (1.06-2.30)* | 1.02 (0.51-2.04) | 1.07 (0.70-1.65) | 0.43 (0.17-1.07) | 1.07 (0.70-1.65) | 0.43 (0.08-2.34) |
|  | 41-50 yr. | 1.42 (0.99-2.05) | 1.48 (0.77-2.85) | 1.06 (0.71-1.57) | 0.88 (0.40-1.93) | 1.06 (0.71-1.57) | 0.88 (0.33-2.37) |
|  | 51-65 yr. | 1 | 1 | 1 | 1 | 1 | 1 |
| Ethnicity | Dutch native | 0.78 (0.58-1.04) | 0.80 (0.48-1.33) | 0.81 (0.58-1.12) | 0.74 (0.38-1.42) | 0.81 (0.58-1.13) | 0.74 (0.39-1.38) |
|  | Non-native | 1 | 1 | 1 | 1 | 1 | 1 |
| Marital status | Single | 1.14 (0.78-1.68) | 0.77 (0.40-1.51) |  |  |  |  |
|  | Married/ committed relationship | 0.80 (0.51-1.2) | 0.38 (0.16-0.88)* |  |  |  |  |
|  | Divorced/ widowed | 1 | 1 |  |  |  |  |
| Education | Low | 1 | 1 | 1 | 1 | 1 | 1 |
|  | Mid-Low | 1.09 (0.75-1.60) | 1.02 (0.52-1.97) | 1.32 (0.87-1.98) | 1.99 (0.87-4.59) | 1.32 (0.86-2.00) | 1.99 (0.37-10.68) |
|  | Mid-High | 1.14 (0.77-1.69) | 0.95 (0.48-1.88) | 1.39 (0.90-2.14) | 1.40 (0.57-3.48) | 1.39 (0.90-2.16) | 1.40 (0.53-3.68) |
|  | High | 0.57 (0.34-0.96)* | 0.38 (0.13-1.11) | 0.79 (0.45-1.38) | 0.59 (0.18-2.02) | 0.79 (0.44-1.39) | 0.59 (0.16-2.19) |
| Employment | Yes | 1.28 (0.87-1.88) | 0.62 (0.31-1.25) | 1.54 (1.00-2.39)* | 0.81 (0.34-1.93) | 1.54 (0.99-2.39) | 0.81 (0.33-1.99) |
|  | No | 1 | 1 | 1 | 1 | 1 | 1 |
| Housing | Sheltered housing | 1.48 (1.04-2.11)* | 1.33 (0.66-2.66) | 1.30 (0.88-1.92) | 1.47 (0.60-3.58) | 1.30 (0.87-1.94) | 1.47 (0.39-5.48) |
|  | Family household | 0.80 (0.55-1.14) | 0.40 (0.18-0.90)* | 0.71 (0.48-1.06) | 0.57 (0.23-1.43) | 0.71 (0.48-1.05) | 0.57 (0.24-1.33) |
|  | Single household | 1 | 1 | 1 | 1 | 1 | 1 |
| Urbanity | > 2500 inh./km2 | 0.94 (0.57-1.57) | 0.47 (0.20-1.12) | 0.92 (0.52-1.61) | 0.15 (0.04-0.53)** | 0.92 (0.52-1.61) | 0.15 (0.00-10.16) |
|  | ≤ 2500 inh./km2 | 1 | 1 | 1 | 1 | 1 | 1 |
| Diagnosis | Psychotic disorders | 0.99 (0.71-1.39) | 1.31 (0.71-2.40) | 0.99 (0.68-1.44) | 2.32 (0.96-5.66) | 0.99 (0.68-1.44) | 2.32 (0.45-12.10) |
|  | Mood disorders | 1 | 1 | 1 | 1 | 1 | 1 |
| Social functioning | Poor^#^ | 1.97 (1.41-2.74)** | 1.08 (0.49-2.37) | 1.70 (1.17-2.47)** | 0.87 (0.37-2.06) | 1.70 (1.17-2.47)** | 0.87 (0.39-1.98) |
|  | Moderate to good^##^ | 1 | 1 | 1 | 1 | 1 | 1 |
| Alcohol abuse  past 6 months | Present | 1.87 (1.37-2.53)*** | 0.92 (0.55-1.56) | 1.58 (1.10-2.26)* | 1.10 (0.52-2.35) | 1.58 (1.10-2.27)* | 1.10 (0.55-2.21) |
|  | Absent | 1 | 1 | 1 | 1 | 1 | 1 |
| Drug use past year | Present | 2.41 (1.77-3.27)*** | 1.40 (0.84-2.34) | 1.72 (1.21-2.46)** | 2.32 (1.13-4.78)* | 1.72 (1.21-2.45)** | 2.32 (0.48-11.12) |
|  | Absent | 1 | 1 | 1 | 1 | 1 | 1 |
| Co-morbid PTSD | Present | 1.47 (1.04-2.08)* | 1.83 (1.03-3.25)* | 1.28 (0.86-1.92) | 1.90 (0.84-4.27) | 1.28 (0.86-1.91) | 1.90 (0.53-6.73) |
|  | Absent | 1 | 1 | 1 | 1 | 1 | 1 |
| Childhood neglect | Present | 1.14 (0.84-1.54) | 2.15 (1.22-3.80)** | 1.07 (0.76-1.50) | 1.79 (0.85-3.75) | 1.07 (0.76-1.51) | 1.79 (0.68-4.70) |
|  | Absent | 1 | 1 | 1 | 1 | 1 | 1 |
| Childhood physical abuse | Present | 1.37 (1.03-1.82)* | 2.38 (1.43-3.97)** | 1.13 (0.82-1.56) | 2.13 (1.01-4.48)* | 1.13 (0.82-1.55) | 2.13 (0.95-4.76) |
|  | Absent | 1 | 1 | 1 | 1 | 1 | 1 |
| Childhood sexual abuse | Present | 1.18 (0.87-1.59) | 0.93 (0.55-1.57) |  |  |  |  |
|  | Absent | 1 | 1 |  |  |  |  |
| Violent perpetration past year | Present | 2.14 (1.55-2.95)*** | 1.65 (0.97-2.81) | 1.62 (1.13-2.31)** | 1.43 (0.73-2.79) | 1.62 (1.13-2.30)** | 1.43 (0.68-3.01) |
|  | Absent | 1 | 1 | 1 | 1 | 1 | 1 |
| Dispositional anger | High^¥^ | 1.53 (1.15-2.04)** | 1.68 (1.00-2.82)* | 1.31 (0.95-1.80) | 1.66 (0.78-3.53) | 1.31 (0.95-1.80) | 1.66 (0.42-6.48) |
|  | Low^¥¥^ | 1 | 1 | 1 | 1 | 1 | 1 |
| * p<0.05 ;** p<0.01; *** p<0.001  ^#^ HONOS score > 9; ^##^  HONOS score =< 9  ^¥^ DAR score >51; ^¥¥^ DAR score <=51  ^a^ Comprises burglary, burglary attempt, bike theft, pickpocketing, robbery, theft (other), car theft, theft from car and car vandalism  Sex, urbanity and social functioning are included in the multivariable model irrespective of model building criteria (grey shading) | | | | | | | |
