# Supplementary material for: Determinants of victimization in patients with severe mental illness: results from a nation-wide cross-sectional survey in the Netherlands
Source: Front Psychiatry. 2025 Mar 17;16:1511841. doi: 10.3389/fpsyt.2025.1511841 (PMC11955743; doi:10.3389/fpsyt.2025.1511841)
Supplement: Supplementary file 1 [file DataSheet1.zip › Appendix Figure A.DOCX]

Appendix Figure A: Flowchart of the study recruitment process

23%

57%

43%

92%

8%

75%

Contact

Refusal by mail

Reason for non-response:

No time/not interested/too tired: 84%

Failed appointments: 7%

No experience of victimization: 2%

Too traumatized: 5%

Other: 2%

Interview

Invited

Yes

No

1763

162

1925

Yes

No

10000

763

Yes

No

3336

Reason for non-response:

No time/not interested/too tired: 81%

Interview too long: 7%

No experience of victimization: 6%

Too traumatized: 2%

Other: 4%

25%

647

96%

4%

Data cleaning

Approved

Rejected

440

9560

Yes

77%

No

764

2572

Random sample
