# Supplementary material for: Determinants of victimization in patients with severe mental illness: results from a nation-wide cross-sectional survey in the Netherlands
Source: Front Psychiatry. 2025 Mar 17;16:1511841. doi: 10.3389/fpsyt.2025.1511841 (PMC11955743; doi:10.3389/fpsyt.2025.1511841)
Supplement: Supplementary file 1 [file DataSheet1.zip › Appendix Table A.DOCX]

Appendix Table A: Determinants of personal, property and total crime victimization estimated on the complete imputed dataset (N=956). Results from univariable hurdle regression analyses

|  |  | | Personal crime victimization^a^ | | | | Property crime victimization^b^ | | Crime victimization^c^  (all crime) | | |  |
| --- | --- | --- | --- | --- | --- | --- | --- | --- | --- | --- | --- | --- |
|  |  | | Binomial logit  Prevalence | | Negative binomial count  Number of incidents | | Binomial logit  Prevalence | Negative binomial count  Number of incidents | Binomial logit  Prevalence | Negative binomial count  Number of incidents | |  |
|  |  | | OR (95%CI) | | IRR (95%CI) | | OR (95%CI) | IRR (95%CI) | OR (95%CI) | IRR (95%CI) | |  |
| Sex | Male | | 0.88 (0.63-1.22) | | 5.10 (2.69-9.66)^***^ | | 1.09 (0.81-1.47) | 5.68 (3.41-9.44)^***^ | 0.92 (0.70-1.20) | 3.13 (2.06-4.78)^***^ | |  |
|  | Female | | 1 | | 1 | | 1 | 1 | 1 | 1 | |  |
| Age | 18–30 yr. | | 1.40 (0.79-2.50) | | 0.09 (0.03-0.29)^***^ | | 2.36 (1.46-3.82)^***^ | 0.47 (0.19-1.14) | 1.93 (1.23-3.02)^**^ | 1.72 (1.23-3.02)^**^ | |  |
|  | 31-40 yr. | | 1.79 (1.16-2.75)^**^ | | 0.04 (0.02-0.10)^***^ | | 1.58 (1.07-2.33)^*^ | 1.02 (0.51-2.05) | 1.61 (1.14-2.27)^**^ | 1.51 (1.14-2.27)^**^ | |  |
|  | 41-50 yr. | | 1.50 (0.99-2.27) | | 0.39 (0.16-0.96)^*^ | | 1.48 (1.03-2.14)^*^ | 3.46 (1.80-6.64)^***^ | 1.66 (1.20-2.28)^**^ | 1.24 (1.20-2.28)^**^ | |  |
|  | 51-65 yr. | | 1 | | 1 | | 1 | 1 | 1 | 1 | |  |
| Ethnicity | Dutch native | | 0.88 (0.58-1.12) | | 0.55 (0.28-1.05) | | 0.80 (0.59-1.07) | 1.61 (0.98-2.64) | 0.91 (0.70-1.18) | 1.02 (0.67-1.57) | |  |
|  | Non-native | | 1 | | 1 | | 1 | 1 | 1 | 1 | |  |
| Marital status | Single | | 0.98 (0.64-1.52) | | 2.58 (1.10-6.06)^*^ | | 1.11 (0.75-1.63) | 0.94 (0.48-1.84) | 1.18 (0.83-1.67) | 2.21 (1.25-3.91)^**^ | |  |
|  | Married/ committed relationship | | 0.90 (0.55-1.49) | | 0.52 (0.20-1.34) | | 0.79 (0.50-1.25) | 1.94 (0.88-4.27) | 0.98 (0.66-1.46) | 0.65 (0.34-11.24) | |  |
|  | Divorced/ widowed | | 1 | | 1 | | 1 | 1 | 1 | 1 | |  |
| Education | Low | | 1 | | 1 | | 1 | 1 | 1 | 1 | |  |
|  | Mid-Low | | 0.93 (0.60-1.42) | | 0.26 (0.11-0.62)^**^ | | 1.13 (0.77-1.64) | 2.15 (1.12-4.13)^*^ | 0.93 (0.60-1.42) | 0.26 (0.11-0.62)^**^ | |  |
|  | Mid-High | | 0.78 (0.49-1.23) | | 0.11 (0.04-0.26)^***^ | | 1.07 (0.72-1.59) | 1.43 (0.72-2.85) | 0.78 (0.49-1.23) | 0.11 (0.04-0.26)^***^ | |  |
|  | High | | 0.92 (0.55-1.56) | | 1.02 (0.34-3.08) | | 0.58 (0.35-0.96)^*^ | 0.45 (0.11-1.85) | 0.92 (0.55-1.56) | 1.02 (0.34-3.08) | |  |
| Employment | Yes | | 1.12 (0.72-1.74) | | 2.32 (0.92-5.86) | | 1.29 (0.87-1.90) | 0.37 (0.18-0.74)^**^ | 1.37 (0.96-1.97) | 1.04 (0.60-1.83) | |  |
|  | No | | 1 | | 1 | | 1 | 1 | 1 | 1 | |  |
| Housing | Sheltered housing | | 1.30 (0.86-1.98) | | 2.82 (1.26-6.29)^*^ | | 1.51 (1.07-2.15)^*^ | 1.10 (0.59-2.09) | 1.30 (0.86-1.98) | 2.82 (1.26-6.29)^**^ | |  |
|  | Family household | | 0.72 (0.46-1.14) | | 0.33 (0.15-0.75)^**^ | | 0.78 (0.54-1.12) | 1.99 (1.05-3.76)^*^ | 0.72 (0.46-1.14) | 0.33 (0.15-0.75)^**^ | |  |
|  | Single household | | 1 | | 1 | | 1 | 1 | 1 | 1 | |  |
| Urbanity | > 2500 inh./km2 | | 1.08 (0.58-2.01) | | 2.90 (0.89-9.43) | | 0.87 (0.51-1.47) | 0.13 (0.05-0.33)^***^ | 0.89 (0.49-1.60) | 1.06 (0.41-2.72) | |  |
|  | ≤ 2500 inh./km2 | | 1 | | 1 | | 1 | 1 | 1 | 1 | |  |
|  |  | |  | |  | |  |  |  |  | |  |
| Diagnosis | Psychotic disorders | | 0.70 (0.48-1.00) | | 4.66 (2.36-9.20)^***^ | | 0.95 (0.68-1.33) | 0.45 (0.25-0.79)^**^ | 0.79 (0.58-1.07) | 2.87 (1.79-4.60)^***^ | |  |
|  | Mood disorders | | 1 | | 1 | | 1 | 1 | 1 | 1 | |  |
| Social functioning | Poor^#^ | | 2.15 (1.34-3.45)^**^ | | 11.52(5.89-22.54)^***^ | | 1.75 (1.19-2.56)^**^ | 1.71 (0.78-3.78) | 2.02 (1.52-2.68)^***^ | 2.48 (1.12-5.49)^*^ | |  |
|  | Moderate to good^##^ | | 1 | | 1 | | 1 | 1 | 1 | 1 | |  |
| Alcohol abuse  past 6 months | Present | | 1.65 (1.17-2.33)^**^ | | 0.81 (0.41-1.56) | | 1.81 (1.33-2.46)^***^ | 0.52 (0.31-0.86)^*^ | 1.80 (1.35-2.41)^***^ | 0.57 (0.37-0.88)^*^ | |  |
|  | Absent | | 1 | | 1 | | 1 | 1 | 1 | 1 | |  |
| Drug use past year | Present | | 2.50 (1.78-3.51)^***^ | | 0.77 (0.40-1.48) | | 2.43 (1.79-3.31)^***^ | 0.69 (0.42-1.13) | 2.78 (2.06-3.75)^***^ | 0.61 (0.40-0.94)^*^ | |  |
|  | Absent | | 1 | | 1 | | 1 | 1 | 1 | 1 | |  |
| Co-morbid PTSD | Present | | 1.38 (0.94-2.04) | | 1.74 (0.80-3.78) | | 1.52 (1.08-2.15)^*^ | 0.99 (0.56-1.74) | 1.37 (0.99-1.86) | 1.12 (0.67-1.86) | |  |
|  | Absent | | 1 | | 1 | | 1 | 1 | 1 | 1 | |  |
| Childhood neglect | Present | | 1.22 (0.86-1.73) | | 0.45 (0.22-0.91)^*^ | | 1.15 (0.85-1.55) | 3.72 (2.11-6.55)^***^ | 1.31 (1.00-1.72) | 1.15 (0.73-1.81) | |  |
|  | Absent | | 1 | | 1 | | 1 | 1 | 1 | 1 | |  |
| Childhood physical abuse | Present | | 2.11 (1.52-2.92)^***^ | | 1.15 (0.60-2.21) | | 1.39 (1.04-1.84)^*^ | 1.28 (0.78-2.07) | 1.69 (1.30-2.19)^***^ | 2.84 (1.87-4.31)^***^ | |  |
|  | Absent | | 1 | | 1 | | 1 | 1 | 1 | 1 | |  |
| Childhood sexual abuse | Present | | 2.16 (1.55-3.00)^***^ | | 1.19 (0.63-2.29) | | 1.15 (0.85-1.55) | 0.50 (0.30-0.85)^*^ | 1.71 (1.30-2.26)^***^ | 0.88 (0.57-1.36) | |  |
|  | Absent | | 1 | | 1 | | 1 | 1 | 1 | 1 | |  |
| Violent perpetration past year | Present | | 3.01 (2.12-4.28)^***^ | | 0.21 (0.11-0.40)^***^ | | 2.14 (1.54-2.96)^***^ | 0.86 (0.51-1.46) | 2.44 (1.78-3.35)^***^ | 0.42 (0.27-0.65)^***^ | |  |
|  | Absent | | 1 | | 1 | | 1 | 1 | 1 | 1 | |  |
| Dispositional anger | High^¥^ | | 1.77 (1.26-2.47)^***^ | | 1.31 (0.67-2.56) | | 1.48 (1.11-1.97)^**^ | 0.84 (0.51-1.38) | 1.49 (1.15-1.93)^**^ | 2.48 (1.63-3.78)^***^ | |  |
|  | Low^¥¥^ | | 1 | | 1 | | 1 | 1 | 1 | 1 | |  |
| * p<0.05 ;** p<0.01; *** p<0.001 | | | | | | | | | | |  |  |
| ^#^ HONOS score > 9; ^##^  HONOS score =< 9  ^¥^ DAR score >51; ^¥¥^ DAR score <=51  ^a^ Comprises sexual harassment or assault, threatened with violence, and physical assault  ^b^ Comprises burglary, burglary attempt, bike theft, pickpocketing, robbery, theft (other)  ^c^ Comprises burglary, burglary attempt, bike theft, pickpocketing, robbery, theft (other), vandalism (other), sexual harassment or assault, threatened with violence, physical assault, crime (other) | | | | | | | | | | | | |
|  | |  | |  | |  |  |  |  |  |  |  |
